# Central and Direct Regulation of Testicular Activity by Gonadotropin-Inhibitory Hormone and Its Receptor

**DOI:** 10.3389/fendo.2014.00008

**Published:** 2014-01-27

**Authors:** Takayoshi Ubuka, You Lee Son, Yasuko Tobari, Misato Narihiro, George E. Bentley, Lance J. Kriegsfeld, Kazuyoshi Tsutsui

**Affiliations:** ^1^Department of Biology, Center for Medical Life Science, Waseda University, Tokyo, Japan; ^2^Department of Integrative Biology, Helen Wills Neuroscience Institute, University of California at Berkeley, Berkeley, CA, USA; ^3^Department of Psychology, Helen Wills Neuroscience Institute, University of California at Berkeley, Berkeley, CA, USA

**Keywords:** gonadotropin-inhibitory hormone, GPR147, gonadotropins, testosterone, spermatogenesis, melatonin, stress, social environment

## Abstract

Gonadotropin-inhibitory hormone (GnIH) was first identified in Japanese quail to be an inhibitor of gonadotropin synthesis and release. GnIH peptides have since been identified in all vertebrates, and all share an LPXRFamide (X = L or Q) motif at their C-termini. The receptor for GnIH is the G protein-coupled receptor 147 (GPR147), which inhibits cAMP signaling. Cell bodies of GnIH neurons are located in the paraventricular nucleus (PVN) in birds and the dorsomedial hypothalamic area (DMH) in most mammals. GnIH neurons in the PVN or DMH project to the median eminence to control anterior pituitary function via GPR147 expressed in gonadotropes. Further, GnIH inhibits gonadotropin-releasing hormone (GnRH)-induced gonadotropin subunit gene transcription by inhibiting the adenylate cyclase/cAMP/PKA-dependent ERK pathway in an immortalized mouse gonadotrope cell line (LβT2 cells). GnIH neurons also project to GnRH neurons that express GPR147 in the preoptic area (POA) in birds and mammals. Accordingly, GnIH can inhibit gonadotropin synthesis and release by decreasing the activity of GnRH neurons as well as by directly inhibiting pituitary gonadotrope activity. GnIH and GPR147 can thus centrally suppress testosterone secretion and spermatogenesis by acting in the hypothalamic–pituitary–gonadal axis. GnIH and GPR147 are also expressed in the testis of birds and mammals, possibly acting in an autocrine/paracrine manner to suppress testosterone secretion and spermatogenesis. GnIH expression is also regulated by melatonin, stress, and social environment in birds and mammals. Accordingly, the GnIH–GPR147 system may play a role in transducing physical and social environmental information to regulate optimal testicular activity in birds and mammals. This review discusses central and direct inhibitory effects of GnIH and GPR147 on testosterone secretion and spermatogenesis in birds and mammals.

## Introduction

**Figure 1:**
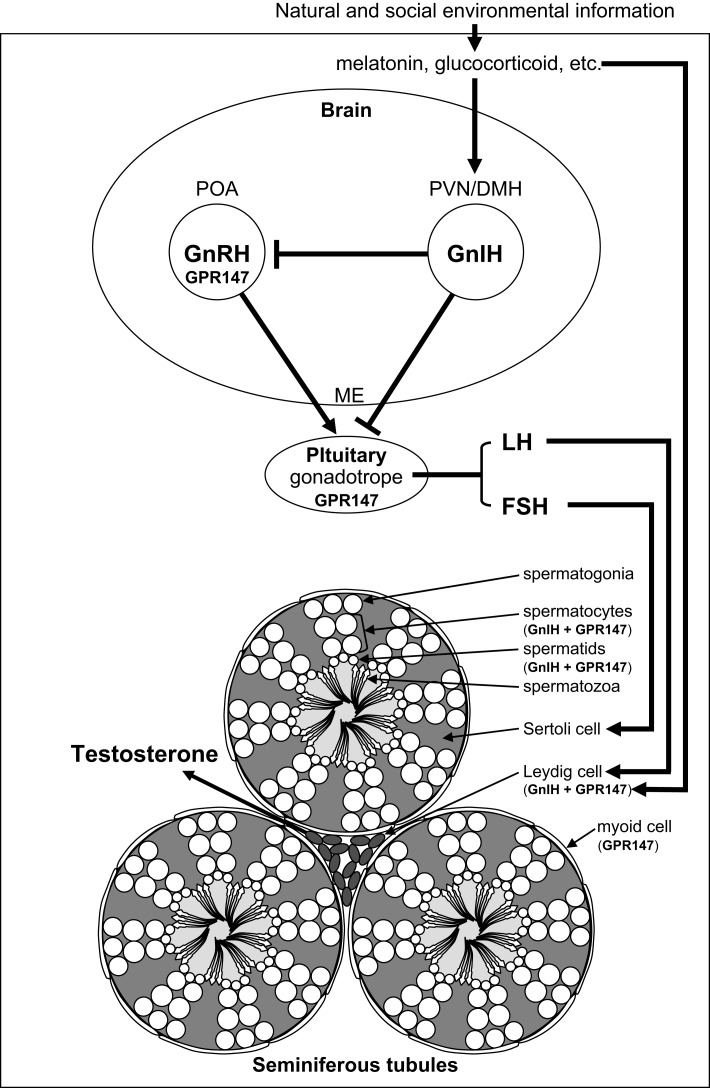
**Schematic model of central and direct actions of GnIH on testicular activity in birds and mammals**. Neuronal cell bodies expressing gonadotropin-inhibitory hormone (GnIH) are located in the paraventricular nucleus (PVN) in birds and the dorsomedial hypothalamic area (DMH) in mammals. GnIH neurons in the PVN or DMH project to the median eminence (ME) to control anterior pituitary function via GnIH receptor (GPR147) expressed in gonadotropes. GnIH neurons also project to gonadotropin-releasing hormone (GnRH) neurons that express GPR147 in the preoptic area (POA) in birds and mammals. Accordingly, GnIH may inhibit gonadotropin [luteinizing hormone (LH) and follicle-stimulating hormone (FSH)] synthesis and release by decreasing the activity of GnRH neurons as well as directly inhibiting pituitary gonadotrope function. GnIH and/or GPR147 are also expressed in the testis of birds (3, 4) and mammals (5), possibly acting in an autocrine/paracrine manner to suppress testosterone secretion and spermatogenesis. GnIH and GPR147 can thus suppress testosterone secretion and spermatogenesis by acting at all levels of the hypothalamic–pituitary–testicular axis. GnIH expression is further regulated by melatonin, glucocorticoids, and the social environment in birds and mammals suggesting an important role in appropriate regulation of testicular activity seasonally, during times of stress and when interacting with conspecifics in birds and mammals.

Testicular activity is under the control of the gonadotropins, luteinizing hormone (LH) and follicle-stimulating hormone (FSH), which are synthesized in the anterior pituitary gland. LH and FSH are released into the circulation and activate their receptors expressed on Leydig cells and Sertoli cells, respectively, to stimulate testosterone secretion and spermatogenesis in the testis (1) (Figure 1). Spermatogenesis is a conserved process in vertebrate testis, where spermatogonia develop into spermatocytes that undergo meiosis to produce spermatids that enter spermiogenesis and undergo a morphological transformation into spermatozoa (2) (Figure 1). The process of germ cell development and maturation can be divided into two distinct patterns in vertebrates, one in anamniotes (fish and amphibia) and the other in amniotes (reptiles, birds, and mammals). In anamniotes, spermatogenesis occurs in spermatocysts, which for most species develop in seminiferous lobules. In amniotes, spermatogenesis occurs in seminiferous tubules that possess a permanent population of Sertoli cells, which support spermatogenesis and spermiogenesis, and spermatogonia, and act as a germ cell reservoir for succeeding bouts of spermatogenic activity (2) (Figure 1).

The hypothalamic decapeptide gonadotropin-releasing hormone (GnRH) is the primary factor that regulates gonadotropin secretion. GnRH is produced in the preoptic area (POA) and released at the median eminence to stimulate gonadotropin secretion from the pituitary (Figure 1). GnRH was first identified in mammals (6, 7) and subsequently in birds (8, 9) and other vertebrates. Testicular steroids and inhibin can modulate gonadotropin secretion by negative feedback. Although dopamine has been reported as an inhibitor of gonadotropin secretion in several fishes (10), no hypothalamic neuropeptide inhibitor of gonadotropin secretion was known in vertebrates. In 2000, a hypothalamic neuropeptide was shown to inhibit gonadotropin release from the cultured quail anterior pituitary gland and it was named gonadotropin-inhibitory hormone [GnIH; (11)] (Figure 1). GnIH was originally identified in birds (11) and subsequently in various vertebrates including mammals [for reviews, see Ref. (12–21)] (Table 1). Based on extensive studies on birds and mammals, it appeared that GnIH can inhibit gonadotropin secretion by decreasing the activity of GnRH neurons as well as directly inhibiting pituitary gonadotropes [for reviews, see Ref. (12–21)]. GnIH and its receptor (GPR147) are also expressed in the gonads of birds (3, 4, 22, 23) and mammals (5, 24–26) including humans (27), possibly acting in an autocrine/paracrine manner (Figure 1). This review summarizes possible central and direct effects of GnIH and GPR147 on testosterone secretion and spermatogenesis in birds and mammals.

**Table 1:** **Amino acid sequences of avian and mammalian GnIHs [LPXRFamide (X = L or Q) peptides]**.

	Animal	Name	Sequence	Reference
Birds	Quail	GnIH	SIKPSAY**LPLRFa**	Tsutsui et al. (11)
		GnIH-RP-1^a^	SLNFEEMKDWGSKNFMKVNTPT VNKVPNSVAN**LPLRFa**	Satake et al. (28)
		GnIH-RP-2	SSIQSLLN**LPQRFa**	Satake et al. (28)
	Chicken	GnIH^a^	SIRPSAY**LPLRFa**	Ikemoto et al. (29)
		GnIH-RP-1^a^	SLNFEEMKDWGSKNFLKVNTPT VNKVPNSVAN**LPLRFa**	Ikemoto et al. (29)
		GnIH-RP-2^a^	SSIQSLLN**LPQRFa**	Ikemoto et al. (29)
	Sparrow	GnIH^a^	SIKPFSN**LPLRFa**	Osugi et al. (30)
		GnIH-RP-1^a^	SLNFEEMEDWGSKDIIKMNPF TASKMPNSVAN**LPLRFa**	Osugi et al. (30)
		GnIH-RP-2^a^	SPLVKGSSQSLLN**LPQRFa**	Osugi et al. (30)
	Starling	GnIH	SIKPFAN**LPLRFa**	Ubuka et al. (31)
		GnIH-RP-1^a^	SLNFDEMEDWGSKDIIKMNPFT VSKMPNSVAN**LPLRFa**	Ubuka et al. (31)
		GnIH-RP-2^a^	GSSQSLLN**LPQRFa**	Ubuka et al. (31)
	Zebra finch	GnIH	SIKPFSN**LPLRFa**	Tobari et al. (32)
		GnIH-RP-1^a^	SLNFEEMEDWRSKDIIKMNPF AASKMPNSVAN**LPLRFa**	Tobari et al. (32)
		GnIH-RP-2^a^	SPLVKGSSQSLLN**LPQRFa**	Tobari et al. (32)
Mammals	Human	RFRP-1	MPHSFAN**LPLRFa**	Ubuka et al. (33)
		RFRP-3	VPN**LPQRFa**	Ubuka et al. (33)
	Macaque	RFRP-1^a^	MPHSVTN**LPLRFa**	Ubuka et al. (34)
		RFRP-3	SGRNMEVSLVRQVLN**LPQRFa**	Ubuka et al. (34)
	Bovine	RFRP-1	SLTFEEVKDWAPKIKMNKPV VNKMPPSAAN**LPLRFa**	Fukusumi et al. (35)
		RFRP-3	AMAHLPLRLGKNREDSLS RWVPN**LPQRFa**	Yoshida et al. (36)
	Ovine	RFRP-1^a^	SLTFEEVKDWGPKIKMNT PAVNKMPPSAAN**LPLRFa**	Clarke et al. (37)
		RFRP-3^a^	VPN**LPQRFa**	Clarke et al. (37)
	Rat	RFRP-1^a^	SVTFQELKDWGAKKDIKMS PAPANKVPHSAAN**LPLRFa**	Ukena et al. (38)
		RFRP-3	ANMEAGTMSHFPS**LPQRFa**	Ukena et al. (38)
	Hamster	RFRP-1	SPAPANKVPHSAAN**LPLRFa**	Ubuka et al. (39)
		RFRP-3	TLSRVPS**LPQRFa**	Ubuka et al. (39)

*^a^Putative peptides. The C-terminal LPXRFamide (X = L or Q) motifs are shown in bold*.

## GnIH Receptor and Cell Signaling

Bonini et al. (40) have identified two G protein-coupled receptors (GPCRs) for neuropeptide FF (NPFF), which has a PQRFamide motif at its C-terminus, and named them as NPFF1 (identical to GPR147) and NPFF2 (identical to GPR74). Hinuma et al. (41) have reported a specific receptor for mammalian GnIH, RFamide-related peptide (RFRP), and named it OT7T022, which was identical to NPFF1 (GPR147). The binding affinities for GPR147 and GPR74 and the signal transduction pathway were examined, using various analogs of GnIHs (RFRPs) and NPFF. RFRPs showed a higher affinity for GPR147, whereas NPFF had potent agonistic activity for GPR74 (40, 42). Accordingly, GPR147 (NPFF1, OT7T022) was suggested to be the principal receptor for GnIH (RFRP). It was also shown that GnIHs (RFRPs) suppress cAMP production in Chinese hamster ovarian cells transfected with GPR147 cDNA, suggesting that GPR147 couples to G_αi_ protein (41).

Yin et al. (43) identified GnIH receptor (GPR147) in the quail diencephalon and characterized its binding activity. First, a cDNA encoding a putative *GPR147* was cloned using PCR primers designed from the sequence of the receptor for RFRPs. The crude membrane fraction of COS-7 cells transfected with the putative *GPR147* cDNA specifically bound GnIH, GnIH-related peptides (-RPs), and RFRPs, which have an LPXRFamide (X = L or Q) motif at their C-termini, in a concentration-dependent manner (43). In contrast, C-terminal non-amidated GnIH failed to bind the receptor. Accordingly, the C-terminal LPXRFamide (X = L or Q) motif seems to be critical for its binding to GPR147 (43). It was suggested that there is no functional difference among GnIH and GnIH-RPs because GPR147 bound GnIH and GnIH-RPs with similar affinities (43). Further studies are required to investigate if GnIH and GnIH-RPs work additively or synergistically to achieve their effects on the target cells that express GnIH-R.

Ikemoto and Park (29) cloned *GnIH, GPR147*, and *GPR74* cDNAs in the chicken. *GPR147* cDNA was expressed only in the brain and pituitary, where GnIH may act directly on gonadotropes. On the other hand, *GPR74* cDNA was ubiquitously expressed in various tissue and organs where GnIH action is unknown. Quail GnIH and putative chicken GnIH inhibited G_αi2_ mRNA expression in COS-7 cells transiently transfected with chicken *GPR147* or *GPR74*. However, the effect of GnIHs on the inhibition of G_αi2_ mRNA expression in COS-7 cells was about 100-fold stronger in COS-7 cells transfected with *GPR147* than *GPR74* (29). These results further suggest that GPR147 is the principal receptor for GnIH in birds as in mammals.

To further investigate the intracellular signaling pathway responsible for the actions of GnIH and its possible interaction with GnRH, Son et al. (44) used a mouse gonadotrope cell line, LβT2. Using this cell line, this group established that mouse GnIHs (mRFRPs) effectively inhibit GnRH-induced cAMP signaling, indicating that mouse GnIHs (mRFRPs) function as inhibitors of adenylate cyclase (AC). They further showed that mouse GnIHs (mRFRPs) inhibit GnRH-stimulated ERK phosphorylation and gonadotropin subunit gene transcription. The results indicated that mouse GnIHs (mRFRPs) inhibit GnRH-induced gonadotropin subunit gene transcriptions by inhibiting AC/cAMP/PKA-dependent ERK activation in LβT2 cells (44).

Shimizu and Bédécarrats (45) showed that *GPR147* mRNA levels fluctuate in an opposite manner to GnRH-receptor-III, a pituitary specific form of GnRH receptor (GnRH-R), in the chicken (46, 47) according to reproductive stages. They demonstrated that the chicken GPR147 inhibits cAMP production, most likely by coupling to G_αi_. This inhibition significantly reduces GnRH-induced cAMP responsive element activation in a dose-dependent manner, and the ratio of GnRH/GnIH receptors was a significant modulatory factor. From these results they proposed that in avian species, sexual maturation is characterized by a change in GnIH/GnRH receptor ratio, changing pituitary sensitivity from GnIH inhibition of, to GnRH stimulation of, gonadotropin secretion (45).

## Suppression of Testicular Activity by GnIH Inhibition of Gonadotropin Secretion

Gonadotropin-inhibitory hormone precursor mRNA was first localized by Southern blot analysis of the RT-PCR products in the quail brain. Within the samples from telencephalon, diencephalon, mesencephalon, and cerebellum, GnIH precursor mRNA was only expressed in the diencephalon (28). *In situ* hybridization for GnIH precursor mRNA showed that cells expressing *GnIH* mRNA are clustered in the paraventricular nucleus (PVN) in the hypothalamus (48). Immunohistochemistry using an antibody raised against avian GnIH has revealed that GnIH-ir neurons are clustered in the PVN in quail and other birds (11, 30–32, 49, 50) (Figure 1).

In mammals, GnIH (RFRP) precursor mRNA is expressed in the dorsomedial hypothalamic area (DMH) in mouse and hamster brains, as visualized by *in situ* hybridization (39, 51) (Figure 1). Mammalian GnIH (RFRP) precursor mRNA is expressed in the periventricular nucleus (PerVN), and in the area between the dorsomedial nucleus (DMN) and the ventromedial nucleus (VMN) of the hypothalamus in the rat brain (41, 52). *GnIH (RFRP)* mRNA expressing neuronal cell bodies are localized in the intermediate periventricular nucleus (IPe) of the hypothalamus in the macaque (34), and in the DMN and PVN in the sheep (37).

Immunohistochemical studies using light and confocal microscopy showed that GnIH (RFRP)-ir axon terminals are in close contact with GnRH neurons in birds (50), rodents (39, 51), monkeys (34), and humans (33) (Figure 1), suggesting direct inhibition of GnRH cells by GnIH. Ubuka et al. (31) investigated the interaction of GnIH neuronal fibers with GnRH neurons in the European starling brain. Birds possess at least two forms of GnRH in their brains. One form is GnRH1 which is thought to be released at the median eminence to stimulate the secretion of gonadotropins from the anterior pituitary (8, 9, 53–57). The second form of GnRH, GnRH2 (58, 59), is thought to influence reproductive behaviors in birds (60) and mammals (61, 62). Double-label immunocytochemistry showed GnIH axon terminals on GnRH1 and GnRH2 neurons in the songbird brain (31, 50, 63) suggesting regulation of both gonadotropin secretion and reproductive behavior. *In situ* hybridization of starling *GPR147* mRNA combined with GnRH immunocytochemistry further showed the expression of *GPR147* mRNA in GnRH1 and GnRH2 neurons (31). Similarly, in Siberian hamsters, double-label immunocytochemistry revealed GnIH axon terminals on GnRH neurons, with a subset of GnRH neurons expressing GPR147 (39). Using immunomagnetic purification of GnRH cells, single-cell nested RT-PCR, and *in situ* hybridization, Rizwan et al. (64) showed that 33% of GnRH neurons expressed GPR147, whereas GPR74 was not expressed in either population in mice.

Central administration of GnIH inhibits the release of gonadotropins in white-crowned sparrows (65), Syrian hamsters (51), rats (66), and Siberian hamsters (39) as does peripheral administration of GnIH (30, 51, 67). Direct application of mouse GnIH (RFRP-3) to GnRH cells in mouse brain slices decreased firing rate in a subpopulation of GnRH cells (68). GnIH (RFRP-3) also inhibited firing of kisspeptin-activated vGluT2 (vesicular glutamate transporter 2)-GnRH neurons as well as of kisspeptin-insensitive GnRH neurons (69). These findings suggest that GnIH may inhibit gonadotropin secretion by decreasing the activity of GnRH neurons in addition to directly regulating pituitary gonadotropes in birds and mammals (Figure 1). Importantly, the inhibitory action of GnIH (RFRP-1 and RFRP-3) was only observed in reproductively active long-day (LD) Siberian hamsters that have high gonadotropin concentration, and GnIH (RFRP-1 and RFRP-3) increased basal gonadotropin concentration in reproductively inactive short-day (SD) hamsters (39).

Given the existence of GnIH-ir fibers at the median eminence in birds (11, 30, 31, 48, 50), much of the work to date has focused on the role of GnIH in pituitary gonadotrope regulation (Figure 1). As indicated previously, GnIH suppresses gonadotropin synthesis and/or release from cultured quail and chicken anterior pituitary gland (11, 70). In mammals, abundant GnIH (RFRP)-ir fibers are observed in the median eminence of sheep (37), macaque (34), hamsters (71), and humans (33). As in birds, mammalian GnIH (RFRP-3) inhibits gonadotropin synthesis and/or release from cultured pituitaries in sheep (72) and cattle (73). Peripheral administration of GnIH (RFRP-3) also inhibits gonadotropin release in sheep (37), rats (74), and cattle (73), suggesting actions on the pituitary. Finally, *GPR147* mRNA is expressed in gonadotropes in the human pituitary (33). Together, these findings suggest that GnIH and RFRP-3 act directly on the pituitary to inhibit gonadotropin secretion, at least in these avian and mammalian species (Figure 1).

Further evidence for a direct action of GnIH on the pituitary comes from a study by Sari et al. (72) where they investigated the effects of GnIH (RFRP-3) on the expression of gonadotropin β-subunit genes in ovine pituitary cells. GnRH or vehicle pulses were given to pituitary cells every 8 h for 24 h with and without GnIH (RFRP-3) treatment. GnIH (RFRP-3) reduced LH and FSH secretion stimulated by GnRH. GnIH (RFRP-3) also reduced GnRH-stimulated LHβ and FSHβ subunit gene expressions. Further, GnIH (RFRP-3) abolished GnRH-stimulated phosphorylation of ERK in the pituitary (72).

To establish whether or not GnIH is endogenously released into the anterior pituitary, Smith et al. (75) directly measured GnIH (RFRP-3) in hypophyseal portal blood in ewes during the non-breeding (anestrous) season and during the luteal and follicular phases of the estrous cycle in the breeding season. Pulsatile GnIH (RFRP-3) secretion was observed in the portal blood, with pulse amplitude and pulse frequency being higher during the non-breeding season. Additionally, the magnitude of the LH response to GnRH was reduced by GnIH (RFRP-3) administration in hypothalamo-pituitary-disconnected ewes, providing support for important functionality of this pathway. Together, these data provide convincing evidence that GnIH (RFRP-3) is secreted into portal blood to act on pituitary gonadotropes, reducing the action of GnRH in sheep (75).

To further establish the functional significance and mode of action of GnIH, Ubuka et al. (67) investigated the role of GnIH on gonadal development and maintenance in male quail. Continuous peripheral administration of GnIH to mature birds via osmotic pumps for 2 weeks decreased the expressions of gonadotropin *common α* and *LHβ* subunit mRNAs in a dose-dependent manner. As expected, plasma LH and testosterone concentrations were also decreased dose dependently. Administration of GnIH to mature birds further induced testicular apoptosis, primarily observed in Sertoli cells, spermatogonia, and spermatocytes, and decreased spermatogenic activity in the testis, either through direct actions of GnIH at the level of the gonads (see below) or through decreased gonadotropin and testosterone concentrations. In immature birds, daily peripheral administration of GnIH for 2 weeks suppressed normal testicular growth and the rise in plasma testosterone concentrations. These results indicate that GnIH inhibits testicular development and maintenance either through decreased gonadotropin synthesis and release or via direct actions on the testes (67) (Figure 1).

## GnIH and GnIH Receptor in the Testis

Vertebrate gonads are known to express many “neuropeptides.” Bentley et al. (3) demonstrated the expression of GnIH and its receptor in the avian reproductive system, including the gonads and accessory reproductive organs of Passeriform and Galliform birds. Binding sites for GnIH were identified via receptor fluorography in the interstitial layer and seminiferous tubules of the testis. Immunocytochemistry detected GnIH in testicular interstitial cells and germ cells, and pseudostratified columnar epithelial cells in the epididymis. *In situ* hybridization for *GPR147* mRNA produced a strong reaction product in the germ cells and interstitium in the testes as well as pseudostratified columnar epithelial cells. The distribution of GnIH and its receptor suggested a potential for autocrine/paracrine regulation of testosterone production and germ cell differentiation and maturation in birds (3) (Figure 1).

To examine the functional significance of these findings, McGuire and Bentley (4) investigated the action of GnIH and GnIH receptor in the testis of house sparrow. GnIH precursor mRNA was expressed in the interstitium and *GPR147* mRNA was expressed in the interstitium and spermatocytes (Figure 1). GnIH significantly decreased the testosterone secretion from gonadotropin-stimulated testis cultures (4), suggesting that *GnIH* and *GPR147* are expressed in Leydig cells to reduce the effect of LH on testosterone secretion in an autocrine/paracrine manner (Figure 1).

To examine the generality of the findings in birds, Zhao et al. (5) examined GnIH (RFRP), GPR147, and GPR74 expression in the testes of Syrian hamsters. GnIH (RFRP) expression was observed in spermatocytes and in round to early elongated spermatids. GPR147 protein was observed in myoid cells in all stages of spermatogenesis, pachytene spermatocytes, maturation division spermatocytes, and in round and late elongated spermatids. GPR74 proteins only appeared in late elongated spermatids. As in birds, these findings suggest a possible autocrine and/or paracrine role for GnIH (RFRP) in Syrian hamster testis, potentially contributing to the differentiation of spermatids during spermiogenesis (5) (Figure 1).

Anjum et al. (76) investigated the changes in GnRH, GnIH, and GnRH-R in the testis from birth to senescence in mice. They found that increased staining of testicular GnRH-R coincided with increased steroidogenic activity during pubertal and adult stages, whereas decreased staining coincided with decreased steroidogenic activity during senescence, suggesting a putative role of GnRH during testicular pubertal development and senescence. The significant decline in GnRH-R during senescence was suggested to be due to a significant increase in GnIH synthesis during senescence. These observations provide new perspectives in the autocrine/paracrine control of testicular activity by GnRH and GnIH (76).

## Regulation of GnIH Gene Expression

### By melatonin

Investigating the regulatory mechanisms of GnIH expression has important implications for understanding the physiological role of the GnIH system. Photoperiodic mammals regulate reproductive activities according to the annual cycle of changes in nocturnal secretion of melatonin (77). Despite the accepted dogma that birds do not use seasonal changes in melatonin secretion to time their reproductive effort (78, 79), there is some evidence that melatonin is involved in the regulation of several seasonal processes, including gonadal activity, gonadotropin secretion, and timing of egg-laying (80–83). Therefore, Ubuka et al. (84) investigated the action of melatonin on the expression of GnIH in quail, a highly photoperiodic bird species. Because the pineal gland and eyes are the major sources of melatonin in quail (85), Ubuka et al. (84) tested the effects of pinealectomy (Px) combined with orbital enucleation (Ex) (Px plus Ex) and melatonin administration on the expression of GnIH precursor mRNA and GnIH peptide. Px plus Ex decreased the expression of GnIH precursor mRNA and the content of mature GnIH peptide in the hypothalamus; melatonin administration caused a dose-dependent increase in GnIH precursor mRNA and GnIH peptide. Additionally, *Mel_1c_* mRNA, a melatonin receptor subtype, was expressed in GnIH-ir neurons in the PVN. Melatonin receptor autoradiography further revealed the binding of melatonin in the PVN. The results suggested that melatonin acts directly on GnIH neurons through its receptor to induce expression of GnIH (84) (Figure 1). In agreement with this possibility, a later study showed that melatonin can stimulate GnIH release from the quail hypothalamus (86).

Opposite action of melatonin on the inhibition of GnIH (RFRP) expression was shown in Syrian and Siberian hamsters, both photoperiodic mammals (39, 87, 88). *GnIH (RFRP)* mRNA levels and the number of GnIH (RFRP)-ir cell bodies were reduced in sexually quiescent Syrian and Siberian hamsters acclimated to SD photoperiod, compared to sexually active animals maintained under LD photoperiod. The photoperiodic effects on GnIH (RFRP) expression were abolished in Px hamsters and injections of LD hamsters with melatonin reduced the expression of GnIH (RFRP) to SD levels (39, 87). There are also reports showing that the expression of GnIH (RFRP) is regulated by melatonin and season in sheep (89, 90) and rats (91). These results demonstrate that as in quail, GnIH (RFRP), expression is photoperiodically modulated via a melatonin-dependent process in mammals (Figure 1).

Given the localization of GnIH in gonadal tissue, McGuire et al. (23) investigated the possibility that melatonin affects sex steroid secretion and GnIH expression in the gonads of European starlings. Starling gonads expressed mRNAs for *GnIH, GPR147*, and melatonin receptors (*Mel_1b_* and *Mel_1c_*). *GnIH* and *GPR147* expression in the testes was relatively low during the breeding season. The expression levels of *Mel_1b_* and *Mel_1c_* were correlated with *GnIH* and *GPR147* expression, and melatonin up-regulated the expression of *GnIH* mRNA in starling gonads before the breeding season. GnIH and melatonin significantly decreased the testosterone secretion from gonadotropin-stimulated testes *in vitro* prior to, but not during, the breeding season. Thus, local inhibition of testosterone secretion appears to be regulated seasonally at the level of the testis by a mechanism involving melatonin and gonadal GnIH in birds (23) (Figure 1).

### By stress

Stress can lead to reproductive dysfunction across vertebrates (92). To explore whether or not stress might act to inhibit reproduction through the GnIH system, Calisi et al. (93) examined the effects of capture-handling stress on GnIH expression in male and female adult house sparrows. More GnIH-positive neurons were observed in fall birds versus those sampled in the spring, and GnIH-positive neurons were increased significantly by capture-handling stress in spring birds. These data imply that stress influences GnIH early during the breeding season, but not after birds have committed to reproduction (93) (Figure 1). McGuire et al. (94) tested the hypothesis that the gonads are directly influenced by stress hormones, showing that physiologically relevant concentrations of corticosterone can directly up-regulate GnIH expression and decrease the testosterone secretion from gonadotropin-stimulated testes prior to the breeding season (Figure 1). These findings suggest that, stress acts on both central and gonadal GnIH cell populations to inhibit reproductive function.

In agreement with the findings in house sparrows, Kirby et al. (95) showed that both acute and chronic immobilization stress lead to an up-regulation of the expression of GnIH (RFRP) in the DMH of adult male rats associated with the inhibition of downstream hypothalamic–pituitary–testicular activity. Adrenalectomy blocked the stress-induced increase in GnIH (RFRP) expression. Immunohistochemistry revealed that 53% of GnIH (RFRP) cells express receptors for glucocorticoids, suggesting that adrenal glucocorticoids act directly on GnIH (RFRP) cells to increase GnIH expression. Together, these data suggest that GnIH is an important integrator of stress-induced suppression of reproductive function (95) (Figure 1).

Son et al. investigated the mechanism by which glucocorticoids influence GnIH gene expression. As in sparrows and rats, *GR* mRNA was expressed in GnIH neurons in the PVN of quail suggesting direct modulation of GnIH in this species. Although acute corticosterone treatment had no effect on *GnIH* mRNA expression, chronic treatment with corticosterone increased *GnIH* mRNA expression in the quail diencephalon. Using a rat GnIH (RFRP)-expressing neuronal cell line, the authors confirmed the co-expression of *GR* mRNA and established that continuous corticosterone treatment increased *GnIH (RFRP)* mRNA expression. They further demonstrated that corticosterone directly regulates *GnIH* gene transcription by recruitment of GR to its promoter at the glucocorticoid responsive element (GRE) (You Lee Son, Takayoshi Ubuka, Narihiro Misato, Yujiro Fukuda, Itaru Hasunuma, Kazutoshi Yamamoto, and Kazuyoshi Tsutsui, unpublished observation) (Figure 1).

### By social interaction

To examine the impact of mating competition on GnIH, Calisi et al. (96) manipulated nesting opportunities for pairs of European starlings and examined brain *GnIH* mRNA and GnIH content as well as GnRH content. By limiting the number of nest boxes and thus the number of social pairing and nesting opportunities, they observed that birds with nest boxes had significantly fewer numbers of GnIH-producing cells than those without nest boxes and this relationship reversed once eggs had been laid. On the other hand, GnRH content did not vary with nest box ownership. These data suggest that GnIH may serve as a modulator of reproductive function in response to social environment (96) (Figure 1).

It is known that the presence of a female bird as well as copulation rapidly decrease plasma testosterone concentrations in male quail (97, 98). Tobari et al. sought to explore the neurochemical mechanism translating social stimuli into reproductive physiology and behavior. They observed that visual presentation of a female quail decreased plasma LH and testosterone concentrations and this effect was likely to be caused by activation of GnIH neurons in the male quail hypothalamus (Yasuko Tobari, You Lee Son, Takayoshi Ubuka, Yoshihisa Hasegawa, Kazuyoshi Tsutsui, unpublished observation) (Figure 1). Together with the findings in starlings, these findings point to a prominent role for GnIH in mediating the impact of social stimuli on the reproductive axis.

## Summary

As described in the present review, GnIH, acting via GPR147, can suppress the testosterone secretion and spermatogenesis by acting at all levels of the hypothalamic–pituitary–gonadal axis of birds and mammals. GPR147 is expressed in GnRH cells, pituitary gonadotropes, and at the level of the testis and studies described herein at the organismal and cell culture levels provide functional evidence for control at each locus. Additionally, GnIH expression is regulated by melatonin, glucocorticoids, and the social environment. Together, these findings highlight a prominent role for GnIH–GPR147 in integrating physical and social environmental information to regulate reproductive activities appropriately in birds and mammals.

## Conflict of Interest Statement

The authors declare that the research was conducted in the absence of any commercial or financial relationships that could be construed as a potential conflict of interest.
